# Vaginal progesterone to prevent preterm delivery among HIV-infected pregnant women in Zambia: A feasibility study

**DOI:** 10.1371/journal.pone.0224874

**Published:** 2020-01-29

**Authors:** Joan T. Price, Winifreda M. Phiri, Bethany L. Freeman, Bellington Vwalika, Jennifer Winston, Chileshe M. Mabula-Bwalya, Helen B. Mulenga, Jeffrey S. A. Stringer

**Affiliations:** 1 Division of Global Women’s Health, Department of Obstetrics and Gynecology, University of North Carolina at Chapel Hill, Chapel Hill, North Carolina, United States of America; 2 Department of Obstetrics and Gynaecology, University of Zambia School of Medicine, Lusaka, Zambia; 3 UNC Global Projects – Zambia, Lusaka, Zambia; 4 Pharmaceutical Society of Zambia, Lusaka, Zambia; Medical College of Wisconsin, UNITED STATES

## Abstract

Antenatal vaginal progesterone (VP) reduces the risk of preterm birth (PTB) in women with shortened cervical length, and we hypothesize that it may also prevent PTB in women with HIV as their primary risk factor. We conducted a pilot feasibility study in Lusaka, Zambia to investigate uptake, adherence, and retention in preparation for a future efficacy trial. This was a double-masked, placebo-controlled, randomized trial of 200mg daily self-administered VP suppository or placebo. Pregnant women with HIV who were initiating or continuing antiretroviral therapy were eligible for participation. Potential participants underwent ultrasound to assess eligibility; we excluded those ≥24 gestational weeks, with non-viable, multiple gestation, or extrauterine pregnancies, with short cervix (<2.0cm), or with prior spontaneous PTB. Participants initiated study product between 20–24 weeks of gestation and continued to 37 weeks (or delivery, if sooner). The primary outcome was adherence (proportion achieving ≥80% study product use), assessed by dye stain assay of returned single-use vaginal applicators. Secondary outcomes with pre-defined feasibility targets were: uptake (≥50% eligible participants enrolled) and retention (≥90% ascertainment of delivery outcomes). We also evaluated preliminary efficacy by comparing the risk of spontaneous PTB <37 weeks between groups. From July 2017 to June 2018, 208 HIV-infected pregnant women were eligible for screening and 140 (uptake = 67%) were randomly allocated to VP (n = 70) or placebo (n = 70). Mean adherence was 94% (SD±9.4); 91% (n = 125/137) achieved overall adherence ≥80%. Delivery outcomes were ascertained from 134 (96%) participants. Spontaneous PTB occurred in 10 participants (15%) receiving placebo and 8 (12%) receiving progesterone (RR 0.82; 95%CI:0.34–1.97). Spontaneous PTB < 34 weeks occurred in 6 (9%) receiving placebo and 4 (6%) receiving progesterone (RR 0.67; 95%CI:0.20–2.67). In contrast to findings from vaginal microbicide studies in HIV-uninfected, non-pregnant women, our trial participants were highly adherent to daily self-administered vaginal progesterone. The study’s *a priori* criteria for uptake, adherence, and retention were met, indicating that a phase III efficacy trial would be feasible.

## Introduction

Preterm birth (PTB) is the most common cause of neonatal death worldwide.[1] The majority of this disease burden is borne by poor countries in South Asia and sub-Saharan Africa, where access to life-saving neonatal care is often limited.[2] Many of these same countries are also affected by high rates of maternal HIV, which is associated with a 50% increased PTB risk, an effect that antiretroviral drug therapy does not appear to militate against.[3]

Antenatal progesterone—an anti-inflammatory hormone administered intramuscularly or vaginally—reduces the risk of PTB in women with prior spontaneous PTB[4] or shortened cervix,[5–7] and is used widely for these indications. HIV infection leads to immune activation and inflammation, both systemically and in the lower genital tract.[8, 9] While antenatal progesterone has been studied in women with a range of other PTB risk factors,[10] its efficacy in pregnancies complicated by HIV alone is unknown. We conducted a pilot randomized, double-masked, placebo-controlled trial of VP to prevent PTB among HIV-infected pregnant women in Zambia. Our overall goal was to gather feasibility data that might inform the design and implementation of a phase III efficacy trial.

## Methods

### Study design

This was a double-masked, placebo-controlled, randomized trial of VP among HIV-infected pregnant women in Lusaka, Zambia. The study was designed and conducted in accordance with the Consolidated Standards of Reporting Trials (CONSORT 2010) Statement[11] and registered with clinicaltrials.gov (NCT02970552). Its primary design has been reported elsewhere.[12]

### Participants

Pregnant women meeting the following criteria were eligible for enrollment: (1) 18 years of age or older; (2) viable intrauterine pregnancy confirmed by ultrasound; (3) screening ultrasound demonstrating gestational age <24 weeks; (4) antibody-confirmed HIV-1 infection; (5) initiating or continuing antiretroviral therapy (ART) in pregnancy; (6) ability and willingness to provide written informed consent; and (7) willingness to adhere to study visit schedule. We excluded women with any of the following: (1) multiple gestation; (2) non-research indication for antenatal progesterone (i.e., prior spontaneous PTB or cervical length ≤20 mm on screening ultrasound); (3) planned or *in situ* cervical cerclage; (4) evidence of threatened abortion, preterm labor, or ruptured membranes at the time of enrollment; (5) planned delivery prior to 37 weeks; (6) major fetal anomaly detected on screening ultrasound; (7) known uterine anomaly; and (8) known or suspected allergy or contraindication to VP or placebo components.

All women provided written informed consent prior to study participation. The study protocol was approved by the University of North Carolina Institutional Review Board, the University of Zambia Biomedical Research Ethics Committee, the Zambian Medicines Regulatory Authority, and the Zambian National Health Research Authority prior to study initiation.

### Procedures

Potential participants were recruited from the antenatal clinics of two public-sector health centers in Lusaka. At the recruitment clinics, community educators conducted group health talks focusing on general antenatal care as well as study eligibility criteria. Interested antenatal attendees were then escorted to the study clinic for further eligibility determination. To pre-screen for eligibility, we reviewed each patient’s medical record and performed an ultrasound as part of standard antenatal care. Informed consent was administered in the participants’ preferred language, English, Bemba, or Nyanja. After consent, we administered a baseline questionnaire, reviewed medical records, and performed a physical exam. We also did confirmatory HIV testing (Alere Determine HIV-1/2, Abbott Diagnostics), and rapid testing for syphilis (SD Bioline Syphilis 3.0, Abbott Diagnostics), hemoglobin, and urinalysis on all participants. Participants counseled on the importance of ART and study nurses facilitated referrals for those not yet receiving treatment. Women screening positive for syphilis, anemia, or abnormal urinalysis were referred for immediate treatment at the on-site antenatal clinic.

During the screening visit, which typically coincided with first presentation to antenatal care, each participant was assigned an estimated date of delivery (EDD) by ultrasound biometry.[13, 14] Study sonographers trained and certified by the Cervical Length Education and Review (CLEAR) program (https://clear.perinatology.org) measured transvaginal cervical length on each participant once between 16 and 24 gestational weeks.

Randomization occurred between 20 ^0^/_7_ and 23 ^6^/_7_ gestational weeks, inclusive. Participants were randomly assigned with equal probability into one of two study groups using a paper-based system of opaque sealed envelopes in a scheme based on random permuted blocks. Micronized progesterone (200mg) and placebo vaginal suppositories were produced by an experienced compounding pharmacy in Chapel Hill, NC and packaged into kits of 20 suppositories prior to being shipped to Zambia. Participants were assigned unique randomization numbers that corresponded to one of either four active or four placebo lot numbers. At randomization and at each follow-up visit, an on-site pharmacist masked to treatment allocation dispensed study product kits from the corresponding blinded lot. All other research staff members with direct participant contact were masked to both treatment allocation and to assigned product lot numbers.

Participants were instructed to begin daily self-administration of study product starting the day of randomization and continue until 36 ^6^/_7_ weeks, membrane rupture, or delivery, whichever occurred first. Study nurses counseled participants on correct study product use at the randomization visit and at each subsequent study visit. Participants received an instructional sheet on correct product use and storage as well as a discreet carrier, applicators for daily use, and plastic bags to facilitate the return of used applicators. Participants were also instructed to complete a dose diary indicating when they administered study product as instructed.

After randomization, participants returned to the study clinic biweekly to replenish their study product supply and for adherence monitoring by dose diary review and collection of used applicators. Laboratory technicians masked to treatment allocation and to the contents of participant dose diaries tested all returned applicators for evidence of vaginal insertion using a validated dye stain assay (DSA).[15, 16] Each single-use vaginal applicator was treated with an inert dilute food dye (0.05% FD&C Blue No.1) that produces a distinctive streaked color pattern when sprayed on polyethylene plastic applicators after vaginal insertion. A senior study nurse trained in DSA performed 100% quality control of all DSA results.

### Outcomes

The primary outcome of this study was the proportion of women with adequate adherence, which we defined as using at least 80% of prescribed study product.[6, 17] Secondary outcomes were: study uptake, retention, and preliminary efficacy. We defined study feasibility *a priori* as the following: (1) at least 50% of eligible participants agree to be enrolled; (2) at least 70% of participants achieve adequate adherence; and (3) at least 90% ascertainment of delivery outcomes (i.e., date of delivery and infant vital status).

### Statistical analysis

We calculated overall adherence as the total number of DSA-positive applicators a participant returned to the clinic divided by the number of days between the date of her randomization and her last antepartum study visit or delivery, whichever was sooner. Per-visit adherence was calculated as DSA-positive applicators returned over the number of days since the previous attended study visit. We defined uptake as the proportion of women (a) meeting initial ultrasound eligibility criteria and (b) successfully screened who were ultimately randomized into the trial. We calculated retention at each study visit as the number of women completing scheduled visits divided by the number of participants still pregnant at the time of those visits. To quantify retention at delivery, we calculated the proportion of women randomized in the trial for whom we were able to ascertain the date of delivery and infant vital status at birth. We evaluated the association of participant demographic and clinical features on adherence and retention in univariate and multivariable regression models.

We pooled participants from both randomization groups for the overall adherence estimate and analyzed adherence between groups to investigate difference by treatment. The proportion adherent was compared between groups with the Wilcoxon rank sum test. To investigate the utility of a pre-randomization placebo run-in period, we used logistic regression to estimate whether adherence at the first visit following randomization (i.e., 2 weeks later) was predictive of adherence >95% over the remaining study period. We described the performance of dose diary estimates of adherence compared to DSA estimates by calculating sensitivity, specificity, positive predictive value, and negative predictive value with corresponding 95% exact binomial confidence intervals (CI).

In addition to our feasibility analysis, we performed secondary analyses of efficacy and safety outcomes including: (a) delivery prior to 37 weeks gestation; (b) delivery prior to 34 weeks gestation; (c) birth weight <2500g; (d) stillbirth; and (e) related adverse events. We undertook unadjusted analyses to calculate the risk ratio of spontaneous preterm birth <37 gestational weeks and spontaneous preterm birth <34 gestational weeks by randomization group via Poisson regression with robust error variance.

## Results

**Fig 1:**
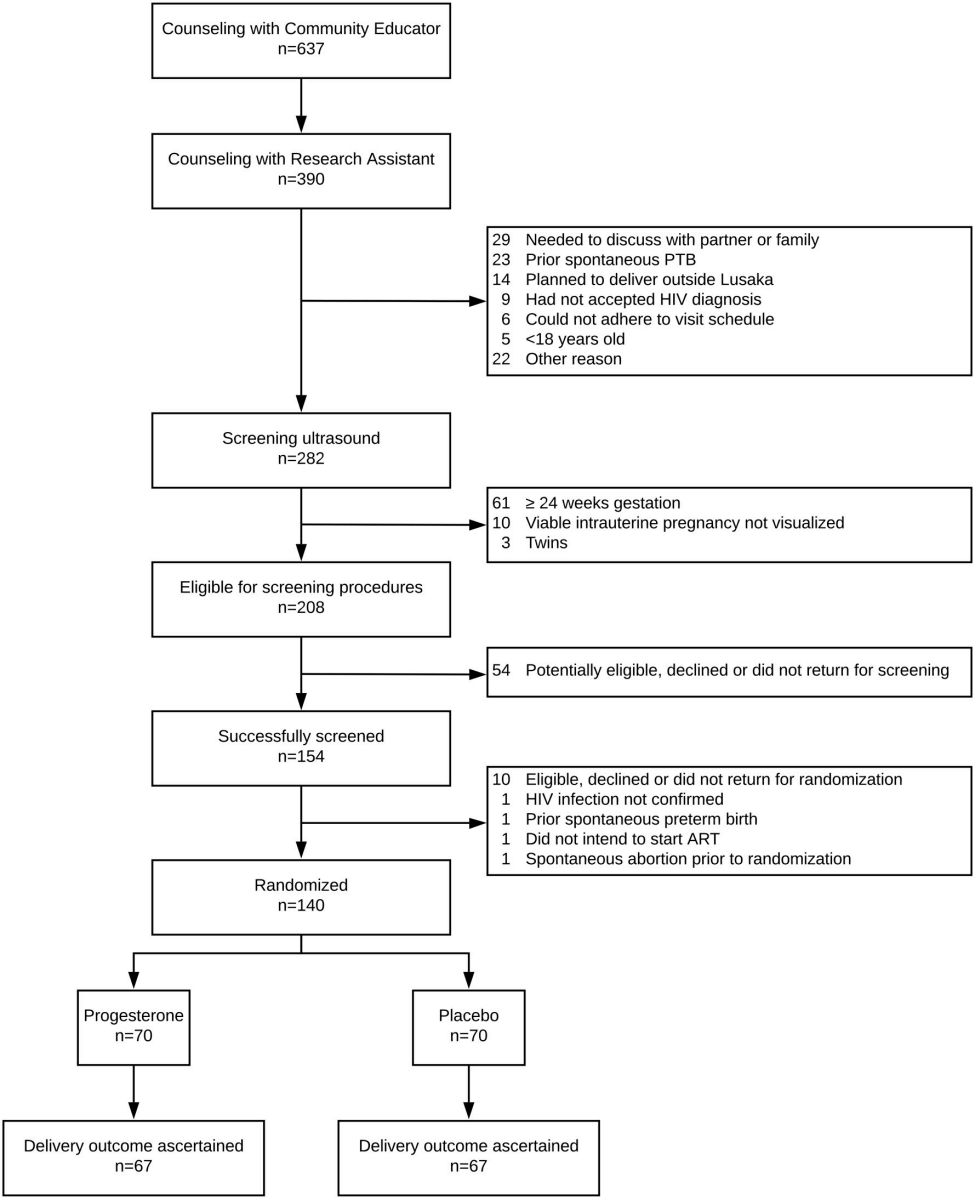
Participant flowchart.

Between July 2017 and June 2018, 140 HIV-infected pregnant women were recruited and randomized at the Kamwala District Health Center in Lusaka (Fig 1). Of 282 women who underwent screening ultrasound, 208 (74%) met ultrasound eligibility criteria. Of these, 154 (74%) successfully completed screening procedures and 140 (67%) were randomized. Because initial accrual was slow (i.e., 18 participants randomized over 3 months), we expanded recruitment in November 2017 to the nearby Chawama First-Level Hospital and achieved a monthly accrual average of 16 participants per month for the remainder of the study.

**Fig 2:**
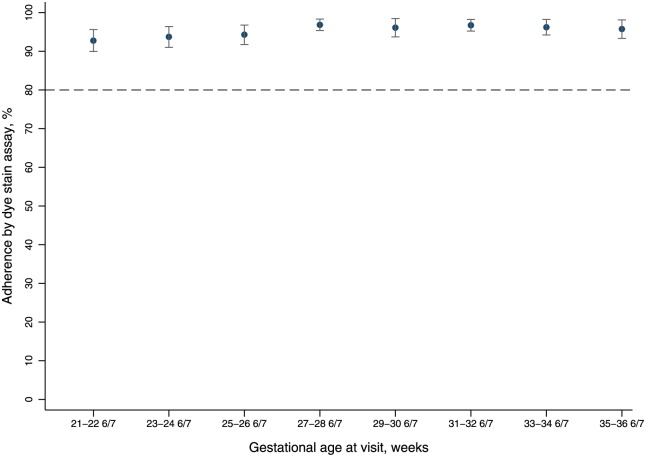
Mean percent adherence by gestational age at visit, n = 137.

Baseline characteristics were similar between participants randomized to progesterone (n = 70) and placebo (n = 70; Table 1). Among the 137 participants (98%) who returned for at least one follow-up study visit, mean adherence to study product was 94.3% (SD±9.4) and exceeded 90% at each study visit (Fig 2). In total, 91% (n = 125/137) of participants achieved overall adherence >80% (Table 2). Adherence was not different in women randomized to progesterone (94.5±9.0%) compared to those randomized to placebo (94.2±9.9%; p = 0.99). Increased adherence at the first study visit following randomization predicted adherence >95% at subsequent visits (OR 1.05; 95% CI 1.02–1.08). Finally, when compared to the gold standard of DSA, self-reported adherence by dose diary demonstrated sensitivity of 99.9% (99.9–100.0%), specificity of 57.1% (95% CI 52.1–61.9%) positive predictive value of 98.5% (98.3–98.7%), and negative predictive value 97.5% (94.6–99.1%) when compared to DSA (Table 3).

**Table 1:** Baseline characteristics of randomized participants, N = 140.

Characteristic	All		Placebo		Progesterone	
	Median (IQR) or N (%)		Median (IQR) or N (%)		Median (IQR) or N (%)	
Age, years	28	25,33	28	25,34	28	24,34
18–24	34	24.3	12	17.1	22	31.4
25–34	82	58.6	45	64.3	37	52.9
≥35	24	17.1	13	18.6	11	15.7
Education, years	8	7,9	8.5	6,9	8	7,9
Did not complete primary	60	42.9	31	44.3	29	41.4
Completed primary	54	38.6	23	32.9	31	44.3
Completed secondary	26	18.6	16	22.9	10	14.3
Marital status						
Neither married nor cohabiting with partner	19	13.6	9	12.9	10	14.3
Either married and/or cohabiting with partner	121	86.4	61	87.1	60	85.7
Running water in house	58	41.4	40	42.9	28	40.0
Electricity in house	124	88.6	65	92.9	59	84.3
Roof material						
Thatch	1	0.7	1	1.4	0	0
Tin	73	52.1	36	51.4	37	52.9
Slate or tile	66	47.1	33	47.1	33	47.1
Cooking fuel						
Electricity	27	19.3	17	24.3	10	14.3
Charcoal / Coal	113	80.7	53	75.7	60	85.7
Toilet facility						
Flush/pour	48	34.3	26	371	22	31.4
Pit/latrine	92	65.7	44	62.9	48	68.6
Household assets	8	5,10	8	5,9	8	6,10
0–4	22	15.7	9	12.9	13	18.6
5–9	83	29.3	47	67.1	36	51.4
≥10	35	25.0	14	20.0	21	30.0
BMI, kg/m2	26.2	24.3,30.1	26.7	24.3,30.1	25.7	24.1,29.7
<18.5	1	0.7	1	1.4	0	0
18.5–30	103	73.6	50	71.4	53	75.7
>30	36	25.7	19	27.1	17	24.3
Parity	2	1,3	2	1,3	2	1,3
Nulliparous	15	10.7	6	8.6	9	12.9
Parous	125	89.3	64	91.4	61	87.1
Hemoglobin, mg/dL	11.6	10.7,12.5	11.6	10.7,12.5	11.6	10.6,12.5
<11	44	31.4	23	32.9	21	30.0
≥11	96	68.6	47	67.1	49	70.0
Timing of HIV diagnosis						
Prior to pregnancy	97	69.3	52	74.3	45	64.3
During pregnancy	43	30.7	18	25.7	25	35.7
Timing of ART initiation						
Prior to pregnancy	95	67.9	50	71.4	45	64.3
During pregnancy	45	32.1	20	28.6	25	35.7
Syphilis screen positive	24	17.1	13	18.6	11	15.7
Urinary tract infection	6	4.3	2	2.9	4	5.7
Alcohol in pregnancy	17	12.1	9	12.9	8	11.4
Tobacco in pregnancy	3	2.0	2	2.9	1	1.4
EGA at screening ultrasound, weeks	19.9	17.3,21.9	19.3	17.0,21.9	20.3	17.4,22.0
<14	10	7.1	3	4.3	7	10.0
EGA at randomization, weeks	20.8	20.0,22.6	20.6	19.9,22.4	20.9	20.1,22.7
Transvaginal cervical length, cm	3.78	3.29,4.19	3.85	3.34,4.21	3.72	3.19,4.18

IQR, interquartile range; ART, antiretroviral therapy; EGA, estimated gestational age

**Table 2:** Adherence based on dye stain assay of returned applicators by gestational age at study visit and by visits since randomization.

	*n*	Overall Mean ± SD	Placebo Mean ± SD	Progesterone Mean ± SD	*p*^a^
**Overall adherence**	137	94.3 (± 9.4)	94.2 (± 9.9)	94.5 (±9.0)	0.986
***By gestational age at study visit***					
21.0–22.6	77	92.8 (±12.5)	92.1 (±11.8)	93.4 (±13.2)	0.305
23.0–24.6	112	93.7 (±14.4)	93.1 (±17.0)	94.4 (±11.1)	0.790
25.0–26.6	131	94.3 (±14.5)	93.8 (±17.8)	94.7 (±10.4)	0.617
27.0–28.6	126	96.9 (±8.4)	95.9 (±10.0)	97.9 (±6.2)	0.253
29.0–30.6	129	96.1 (±13.6)	96.1 (±14.1)	96.1 (±13.3)	0.339
31.0–32.6	123	96.7 (±8.4)	95.6 (±10.6)	97.9 (±5.2)	0.210
33.0–34.6	124	96.2 (±11.3)	97.9 (±5.0)	94.6 (±14.9)	0.228
35.0–36.6	115	95.7 (±12.9)	96.0 (±8.3)	95.5 (±16.4)	0.295
***By follow-up visit since randomization***					
1	137	91.3 (±16.6)	90.5 (±18.9)	92.1 (±13.9)	0.148
2	133	94.9 (±11.6)	93.9 (±13.3)	95.9 (±9.5)	0.108
3	133	95.6 (±13.6)	96.1 (±13.3)	95.0 (±14.1)	0.261
4	132	96.4 (±11.1)	95.7 (±13.7)	97.2 (±7.6)	0.520
5	129	96.4 (±11.2)	96.2 (±10.0)	96.5 (±12.3)	0.554
6	124	96.8 (±9.5)	97.0 (±7.3)	96.7 (±11.3)	0.222
7	92	97.3 (±5.7)	97.3 (±5.8)	97.2 (±5.5)	0.543
8	58	96.3 (±13.6)	97.5 (±4.4)	95.4 (±17.7)	0.676

SD, standard deviation

^**a**^ ***p*** values calculated by Wilcoxon rank-sum

**Table 3:** Performance of dose diary adherence assessment compared to dye stain assay.

	N or % (95% CI)
True positive *(DD+ DSA+)*	11,840
False positive *(DD+ DSA-)*	176
True negative *(DD- DSA-)*	234
False negative *(DD- DSA+)*	6
Sensitivity *Pr(DD+|DSA+)*	99.9% (99.9–100.0%)
Specificity *Pr(DD-|DSA-)*	57.1% (52.1–61.9%)
Positive predictive value *Pr(DSA+|DD+)*	98.5% (98.3–98.7%)
Negative predictive value *Pr(DSA-|DD-)*	97.5% (94.6–99.1%)

CI, confidence interval; DD, dose diary; DSA, dye stain assay; Pr, probability

Baseline covariates associated with higher adherence determined by DSA in multivariable analysis included: having running water at home (coefficient 3.44; 95% CI 0.36–6.54) and parity (coefficient 1.40; 95% CI 0.32–2.48) (Table 4). Receipt of a new HIV diagnosis during the current pregnancy was marginally associated with lower adherence (coefficient -3.28; 95% CI -6.64–0.08).

**Table 4:** Baseline correlates of adherence determined by dye stain assay of returned applicators.

	Univariate			Multivariable ^a^		
Characteristic	coeff	95% CI	*p*	coeff	95% CI	*p*
Age, years	0.30	(0.00–0.60)	0.053	-		
Education, years	0.10	(-0.45–0.64)	0.727			
Running water in house	3.35	(0.15–6.56)	0.040	3.44	(0.36–6.54)	0.029
Electricity in house	3.83	(-1.11–8.77)	0.127	-		
Household assets	0.50	(-0.06–1.07)	0.079			
Parity	1.56	(0.48–2.63)	0.005	1.40	(0.32–2.48)	0.011
BMI, kg/m2	0.25	(-0.05–0.55)	0.097	-		
Hemoglobin, mg/dL	0.33	(-0.88–1.55)	0.587			
HIV diagnosed during pregnancy	-4.31	(-7.71 to -0.93)	0.013	-3.28	(-6.64–0.08)	0.056
ART initiated during pregnancy^b^	-4.17	(-7.52 to -0.82)	0.015	-		
Syphilis	0.47	(-3.73–4.68)	0.824			
Alcohol in pregnancy	1.59	(-3.25–6.43)	0.518			
Tobacco in pregnancy	2.36	(-8.56–13.3)	0.670			
EGA at screening	-0.04	(-0.11–0.02)	0.178			

CI, confidence interval; BMI, body mass index; ART, antiretroviral therapy; EGA, estimated gestational age. Coefficients (coeff) and *p* values of continuous outcome of overall adherence by baseline characteristics calculated by linear regression

^**a**^ Multivariable analysis adjusted for listed variables.

^**b**^ Timing of ART initiation not included in multivariable analysis given collinearity with timing of HIV diagnosis.

Retention was over 90% at each scheduled follow-up study visit (Fig 2). Of the 140 randomized, 119 (85.0%) participants attended all scheduled visits, 14 (10.0%) participants missed 1 scheduled visit, and the remaining 7 (5.0%) missed 2 or more visits. We were able to ascertain delivery outcomes (i.e., at minimum date of delivery and vital status of neonate at delivery) from 134 (96%) participants; 3 women from each randomization group (n = 6) were lost to follow-up. Higher baseline maternal BMI (OR 1.49; 95% CI 1.06–2.08) and higher hemoglobin (OR 1.96; 95% CI 1.00–3.85) were each associated with retention at the delivery visit (Table 5).

**Table 5:** Baseline correlates of participant retention at clinic visits and at the delivery visit.

Characteristic	Retention at clinic visits^a^			Retention at Delivery Visit^b^		
	coeff	95% CI	*p*	OR	95% CI	*p*
Age, years	0.26	(-0.35–0.86)	0.400	1.04	(0.89–1.22)	0.602
Education, years	-0.51	(-1.60–0.57)	0.353	0.81	(0.57–1.13)	0.208
Married or cohabiting	-5.97	(-15.25–3.30)	0.205	-	-	-
Running water in house	-1.64	(-8.12–4.84)	0.617	0.70	(0.14–3.58)	0.665
Electricity in house	2.25	(-7.79–12.28)	0.658	-	-	-
Household assets	0.50	(-0.62–1.63)	0.379	0.93	(0.70–1.25)	0.639
Parity	0.29	(-1.92–2.50)	0.797	1.09	(0.60–1.96)	0.776
BMI, kg/m2	0.32	(-0.27–0.92)	0.283	1.49	(1.06–2.08)	0.020
Hemoglobin, mg/dL	2.20	(-0.18–4.57)	0.070	1.96	(1.00–3.85)	0.051
HIV diagnosed during pregnancy	-1.31	(-8.23–5.61)	0.709	2.28	(0.26–20.1)	0.458
ART initiated during pregnancy	1.34	(-25.58–28.27)	0.922	-	-	-
Syphilis in pregnancy via RPR	1.07	(-7.41–9.54)	0.804	-	-	-
Alcohol in pregnancy	3.84	(-5.93–13.60)	0.439	-	-	-
Tobacco in pregnancy	5.95	(-16.09–27.99)	0.594	-	-	-
EGA at screening	-0.07	(-0.20–0.06)	0.264	0.97	(0.92–1.02)	0.178

OR, odds ratio; CI, confidence interval; BMI, body mass index; ART, antiretroviral therapy; EGA, estimated gestational age

^a^ coefficients and *p* values of proportion retention at clinic visits as continuous outcome calculated via linear regression

^b^ odds ratios and *p* values of retention at delivery visit as dichotomous outcome calculated via logistic regression

Related maternal adverse events, maternal and fetal/neonatal outcomes, and efficacy outcomes were comparable between participants randomized to progesterone and those randomized to placebo (Table 6). Of 134 randomized participants with available delivery data, 19 (14%) delivered before 37 completed gestational weeks, and 11 (8.2%) of those delivered before 34 completed gestational weeks. One preterm delivery prior to 34 weeks was provider-initiated for severe preeclampsia in a participant receiving progesterone; all other preterm deliveries were initiated spontaneously. 10 of 67 (15%) women who received placebo and 8 of 67 (12%) of those who received progesterone delivered spontaneously before 37 weeks of gestation (RR 0.80; 95% CI 0.33–1.91). Spontaneous delivery prior to 34 weeks of gestational age occurred in 6 of 67 (9.0%) women receiving placebo compared to 4 of 67 (6.0%) receiving progesterone (RR 0.67; 95% CI 0.20–2.27). Stillbirth occurred in 4 of 134 (3.0%) pregnancies, 2 receiving placebo and 2 receiving progesterone.

**Table 6:** Frequency of safety and efficacy outcomes by study group.

Outcome	All, *N* = 140	Placebo, *n* = 70	Progesterone, *n* = 70	*P*^b^
	n (%) or median (IQR)	n (%) or median (IQR)	n (%) or median (IQR)	
*Related maternal adverse events*^a^				
Headache	25 (17.9)	10 (14.3)	15 (21.4)	0.270
Nausea / vomiting	12 (8.6)	6 (8.6)	6 (8.6)	1.000
Lower abdominal pain	13 (9.3)	5 (7.1)	8 (11.4)	0.382
Backache	1 (0.7)	1 (1.43)	0	0.316
Diarrhea	4 (2.9)	1 (1.43)	3 (4.3)	0.310
Fatigue / weakness	0	0	0	-
Vaginal itching or burning	13 (9.3)	8 (11.43)	5 (7.1)	0.573
Vaginal discharge	3 (2.14)	1 (1.43)	2 (2.86)	0.559
Urinary tract infection	3 (2.14)	1 (1.43)	2 (2.86)	0.559
*Maternal outcomes*				
Oligo-/polyhydramnios at 32w	0	0	0	-
Gestational hypertension	0	0	0	-
Pre-eclampsia	1 (0.7)	0	1 (1.5)	0.315
Eclampsia	0	0	0	-
Antepartum hemorrhage	0	0	0	-
Preterm prelabor rupture of membranes	0	0	0	-
Cesarean delivery	6 (4.5)	4 (6.0)	2 (3.0)	0.403
Median time to hospital discharge, days	1 (0,1)	1 (0,1)	1 (0,1)	0.413
*Fetal / neonatal outcomes*				
Small for gestational age (n = 128)	35 (27.3)	16 (25.0)	19 (29.7)	0.552
Birthweight <2500g (n = 128)	21 (16.4)	12 (18.8)	9 (14.1)	0.474
Male sex (n = 134)	79 (59.0)	39 (58.2)	40 (59.7)	0.861
Median Apgar score at 1 min (n = 129)	9 (9,9)	9 (9,9)	9 (9,9)	0.456
Median Apgar score at 5 min (n = 129)	9 (9,9)	9 (9,9)	9 (9,9)	0.705
NICU admission (n = 134)	10 (7.5)	7 (10.5)	3 (4.5)	0.189
Early neonatal death (n = 134)	5 (3.9)	3 (4.7)	2 (3.1)	0.636
*Efficacy outcomes* (n = 134)				
Preterm birth <37 weeks	19 (14.2)	10 (14.9)	9 (13.4)	0.804
Spontaneous preterm birth <37 weeks	18 (13.4)	10 (14.9)	8 (11.9)	0.612
Preterm birth <34 weeks	11 (8.2)	6 (9.0)	5 (7.5)	0.753
Spontaneous preterm birth <34 weeks	10 (7.5)	6 (9.0)	4 (6.0)	0.511
Stillbirth	4 (3.0)	2 (3.0)	2 (3.0)	1.000

IQR, inter-quartile range; NICU, neonatal intensive care unit

^**a**^ Signs, symptoms, or diagnoses that started after randomization and were deemed possibly related to study product use

^**b**^ *p* values calculated by chi square or Wilcoxon rank sum for categorical and continuous comparisons, respectively

## Discussion

We present the results of a pilot study evaluating the feasibility of a trial of antenatal vaginal progesterone for the prevention of preterm birth among HIV-infected pregnant women without other major risk factors. The study surpassed its *a priori* goals for trial uptake, study product adherence, and participant retention, indicating that a phase III efficacy trial would be feasible in Zambia. Incidence of adverse events was similar between study groups and, while this study did not have statistical power to investigate efficacy, preliminary efficacy estimates will be used to inform sample size calculations for a full-scale trial.

Participants in both the progesterone and placebo groups had similarly high adherence to study product. The proportion of participants achieving adequate adherence—91%—was comparable to or higher than reported in three other major trials of vaginal progesterone, including one conducted in the UK (94%),[17] and two others that enrolled across multiple international sites (89% and 69%, respectively).[6, 18] In these prior studies, adherence was assessed through participant interviews, dose diaries, and/or counting of unused medication at follow-up visits. One strength of our trial was its use of DSA, a method validated to objectively assess adherence.[15, 19] We also studied participant report as a secondary measure and found dose diary to be a reliable measure of product use when participants were adherent (high sensitivity), but a poor measure in the relatively rare instances of non-adherence (low specificity). However, DSA testing of 100% of returned applicators could be prohibitively resource-intensive in a full-scale trial, which is likely why previous studies of vaginal progesterone have relied on other methods to monitor adherence. We hypothesize that women may have been motivated to insert study product by the knowledge that returned applicators would be tested, so a larger trial might benefit from requesting participants to return all used applicators and then only testing a random subset from each participant to confirm adherence.

A key factor in our decision to undertake a pilot study in Zambia was concern that women may not consistently use a daily vaginal product. Some vaginal microbicide studies in HIV-uninfected, non-pregnant women in sub-Saharan Africa reported substantial discrepancies between self-reported adherence and objective measures such as DSA and plasma drug concentration monitoring.[15, 20] While many factors that may have contributed to low adherence in microbicide trials did not directly apply to our pregnant population already infected with HIV, we did observe some similar associations between study product adherence and participant characteristics such as older age and higher parity.[20, 21] Qualitative interviews in vaginal microbicide trials revealed a number of explanations for low adherence, including a lack of confidence in the efficacy of investigational products, unwanted side effects of vaginal discharge and interference with sexual behavior, and perceived stigmatization associated with using antiretroviral medication despite being HIV-negative.[20, 22] However, altruistic motivations among pregnant women towards their fetuses may have contributed to the high adherence observed in our study, outweighing negative perceptions of research participation or bothersome side effects. Similar findings have been reported to explain higher ART adherence and attendance at clinic visits during pregnancy.[23] Alongside this pilot study, we conducted individual semi-structured interviews with participants to explore the acceptability of participation in the trial, results from which are forthcoming. Another study reported high acceptability of antenatal progesterone—both vaginal and intramuscular formulations—among pregnant women in Zambia,[24] and we anticipate similar findings.

More than half of potentially eligible participants were successfully randomized into this trial, surpassing our target set for uptake. The study team faced initial difficulties in identifying a sufficient number of potentially eligible participants solely from the district clinic where the study was conducted, in response to which we expanded recruitment to a second nearby public-sector facility. This expansion substantially accelerated recruitment and accrual. Despite an accrual velocity that lagged below initial targets, our study demonstrated a similar overall uptake proportion in comparison to other published VP trials.[6, 17, 18]

High participant retention in this study was likely aided by a number of supportive retention efforts. At each study visit, participants were provided transportation reimbursement plus a snack or a meal at longer visits. The value of these reimbursements was approved by the local ethics authority. Study staff placed telephone calls to any participant who missed an appointment, and community staff made home visits to participants unreachable by telephone. We also performed weekly telephone follow-up for participants still pregnant at 40 or more weeks of gestation. Research nurses ascertained key delivery details (i.e., date of delivery and infant vital status) initially over the phone for those who reported having delivered, and encouraged delivered participants to return to the study clinic as soon as possible to review delivery records and ascertain full details of the delivery and subsequent course. The value of social and structural support for improving retention and adherence is well understood,[25] and we hypothesize that biweekly follow-up visits in conjunction with the described supportive measures played an important role in participant retention.

We acknowledge several limitations of the current study. First, participants were recruited from a small catchment area in a single urban setting within Zambia. This geographical focus, as well as other baseline characteristics of our participants, might limit the extent to which trial feasibility could be generalized to studies in other geographical areas, or to younger and primiparous women. Second, while we reported a high positive predictive value of the dose diary (i.e., women with high self-reported adherence by dose diary were likely to return DSA-positive applicators), we note that the specificity of dose diary reporting was much lower (i.e., in instances when the DSA was negative, the dose diary was marked as adherent nearly half of the time). Given the relatively few cases of non-adherence, our study might have missed latent patterns of non-adherence that would occur in a trial with lower overall adherence. However, we suspect that high desirability of successful childbearing in Zambia, request for return of all used applicators, and intensive retention measures will encourage high adherence and retention in a larger trial. Finally, the DSA method itself is not perfect. DSA performance can be affected by the specific dyes and applicator plastics used, and it has been most widely studied in the context of microbicides.[15, 16, 19, 26] Whether the medication being inserted affects the validity of DSA is unknown. DSA positivity does not provide definitive information on whether the suppository (and not just the applicator) was actually inserted. These limitations notwithstanding, our pilot study used a plastic material, dye, and evaluation method that have shown optimal sensitivity and specificity in multiple validation studies.[16, 19]

## Conclusion

This pilot is the first published randomized trial of vaginal progesterone to prevent HIV-related preterm birth. If shown to be effective in a full-scale trial, antenatal progesterone could reduce the high societal and healthcare costs of care for premature infants and of medical and social support for long-term sequelae. Based on high uptake, adherence, and retention in this pilot study, we conclude that a full-scale efficacy trial of vaginal progesterone to prevent preterm birth in HIV-infected gravidas would be feasible in our setting.

## Supporting information

S1 Fig CONSORT Checklist. (PDF)

S2 Fig Study Protocol. (PDF)
