# Supplementary material for: Vaginal progesterone to prevent preterm delivery among HIV-infected pregnant women in Zambia: A feasibility study
Source: PLoS One. 2020 Jan 29;15(1):e0224874. doi: 10.1371/journal.pone.0224874 (PMC6988922; doi:10.1371/journal.pone.0224874)
Supplement: S2 Fig — (PDF) [file pone.0224874.s002.pdf]

A Pilot Study of Vaginal Progesterone to Reduce Preterm Birth among Women Receiving Antiretroviral Therapy in Pregnancy

---

Protocol version 2.1
10 July 2017

TABLE OF CONTENTS **Page**

Investigators and Scientific Team …………………………………………………. 3
Abbreviations List ……………………………………………………………….. 4
Summary……………………………………………………………………….. 5
1.0 Background and Introduction………………………………………………. 6
1.1 Rationale for the study
1.2 Significance
1.3 Aim
1.4 Specific objectives
1.5 Ethical issues
2.0 Literature Review…………………………………………………………. 8
3.0 Methodology…………………………………………………..…………… 9
3.1 Study design
3.2 Study sites and study population
3.3 Study intervention
3.4 Study procedures
3.5 Biological specimen collection
3.6 Laboratory procedures
3.7 Data security and management
3.8 Statistical considerations
3.9 Human participant considerations
3.10 Study monitoring
3.11 Dissemination of findings
4.0 Budget………………………………………………………………………… 21
5.0 Timeframe……………………………………………………………………. 21
6.0 Reference List………………………………………………………………… 21

Abbreviation List

AE	Adverse event
ANC	Antenatal care
ART	Antiretroviral therapy
CI	Confidence interval
DSA	Dye stain assay
FWA	Federal Wide Assurance
HIV	Human immunodeficiency virus
KDC	Kamwala District Clinic
OHRP	Office for Human Research Protections
PrEP	Pre-exposure prophylaxis
PTB	Preterm birth
RCT	Randomized controlled trial
RR	Risk ratio
SAE	Severe adverse event
SSI	Semi-structured interview
UNC	University of North Carolina
VP	Vaginal progesterone

PROTOCOL SUMMARY

Background: Preterm birth (PTB) complicates as many as one in six pregnancies in Zambia. Many infants who are born prematurely die immediately while others face lifelong disability. Maternal HIV is also highly prevalent in Zambia and associated with a doubling of PTB risk. Antiretroviral therapy (ART) in pregnancy appears to increase the risk of PTB even more. Vaginal progesterone (VP) reduces the risk of PTB among women with a prior preterm birth or sonographic evidence of cervical shortening. It is standard of care for prevention of PTB in this high-risk population in the United States. However, VP has not been studied as PTB prophylaxis among HIV-infected women.

Objectives: In preparation for a possible full-scale efficacy trial, this pilot study will evaluate, through quantitative and qualitative methods, whether VP is feasible and acceptable for the prevention of PTB among HIV-infected pregnant women in Lusaka, Zambia.

Design: Single site, mixed method study including a quantitative placebo-controlled, double-masked, pilot randomized controlled trial (RCT) and qualitative semi-structured interviews (SSIs)

Study Arms: Daily self-administered 200 mg micronized vaginal progesterone or indistinguishable placebo randomly allocated in a 1:1 ratio commenced between 20 0/7 and 23 6/7 gestational weeks and continued through 36 $^{6}/_{7}$ gestational weeks, membrane rupture, stillbirth, or delivery, whichever is sooner.

Population: 140 pregnant HIV infected women over the age of 18 seeking antenatal care (ANC) prior to 24 weeks gestation and initiating or continuing antiretroviral therapy in pregnancy will be enrolled in the pilot RCT; an additional 30+ eligible women who decline to participate in the RCT will be invited to participate in SSIs

Study Site: Kamwala District Clinic, Lusaka, Zambia

Duration and Follow up: Women will be enrolled over a period of 5-12 months. Each participant will be followed through delivery.

Endpoints: The pilot RCT will measure rates of enrollment, adherence, and trial retention, as well as preliminary efficacy of the study intervention. Semi-structured interviews will evaluate acceptability of a study of VP to prevent PTB and identify barriers and facilitators to study enrollment, product adherence and study retention.

Relevance: More than 1.5 million HIV-infected women become pregnant each year. Approximately half have access to ART, but all are at increased risk of PTB. VP is a promising and cost-effective intervention to prevent PTB that should be studied in this high-risk population. This pilot study will provide critical insight into the feasibility of a phase III trial by determining whether women are willing to participate, to adhere to study drug, and to complete follow-up.

Statement of Problem: Preterm birth is the most common cause of neonatal death worldwide. Preterm neonates who survive are at an increased risk of short- and long-term morbidity. Women who are HIV-infected have higher than usual chances of delivering their baby prematurely. Given high prevalence of both HIV and preterm birth in Zambia, the prevention of PTB in this high-risk group is a health care priority.

1.0 BACKGROUND AND INTRODUCTION

1.1 RATIONALE

More than 1.5 million HIV-infected women become pregnant each year. Approximately half have access to antiretroviral therapy (ART), but all are at increased risk of preterm birth (PTB). Vaginal progesterone (VP) is a promising and cost-effective intervention to prevent PTB that should be studied in this population.

1.2 SIGNIFICANCE

In Zambia, PTB complicates one in six pregnancies.[1] Many infants who are born too soon die immediately while others face lifelong disability. Maternal HIV is also highly prevalent in Zambia and associated with a near doubling of PTB risk.[2,3] As access to combination ART has become available to an ever-increasing number of pregnant women around the world, perinatal HIV transmission rates have plummeted, but a new complication has emerged. Paradoxically, ART in pregnancy appears to increase the risk of PTB even beyond that attributable to HIV infection itself.[4]

Antenatal VP has been found in randomized trials to reduce the risk of PTB among women with a prior preterm birth or sonographic evidence of cervical shortening.[5-7] VP is standard of care for this latter indication in the United States but has yet to be studied to reduce PTB among HIV-infected women. We hypothesize that VP prophylaxis may reduce PTB among women receiving ART in pregnancy and propose a pilot study to determine whether a full-scale trial would be feasible. We are particularly interested in knowing the extent to which women will adhere to VP (i.e., what proportion of participants will consistently self-administer daily study product as directed), given recently reported challenges to adherence in vaginal microbicide and PrEP trials.

1.3 AIM

We will perform a mixed-method pilot study in Lusaka, Zambia to assess (1) the feasibility of a randomized, placebo-controlled trial of VP for the prevention of PTB among HIV-infected women receiving ART in pregnancy and (2) the acceptability of a trial of VP in this population. Our overall objective is to apply the learning from the quantitative and qualitative components of this study to inform strategies for optimizing enrollment, adherence, and retention in a future phase III efficacy trial.

1.4 SPECIFIC OBJECTIVES

Primary objective

To determine the proportion of study participants who achieve adequate adherence.

Secondary objectives

- Uptake: To determine the proportion of eligible HIV-infected pregnant women who agree to study participation and who are randomized.
- Retention: To determine the proportion of enrolled study participants who complete the study.

- Acceptability: To assess acceptability of a randomized clinical trial of daily VP to prevent PTB among women agreeing to trial participation and among those who choose not to participate.

Tertiary objectives

- To assess whether the use of a placebo run-in period would have utility in a future phase III study.
- To assess safety and preliminary efficacy of VP by comparing PTB rates, adverse maternal outcomes, side effects, and adverse neonatal outcomes between the randomized groups.

1.5 STUDY OUTCOMES

Primary outcomes

Adequate adherence to study product, defined as proper self-administration of at least 80% of prescribed study product doses.

Secondary/tertiary outcomes

- Comparison of self-reported adherence rates, applicator return, and use of applicators measured through a dye stain assay (DSA)
- Enrollment rates among eligible individuals
- Ascertainment of date of delivery and infant vital status at birth
- Reported knowledge, attitudes, and practices related to HIV, ART, risk of PTB, and participation in placebo controlled RCTs, as well as attitudes about medications for the prevention of PTB including acceptability of daily vaginal administration
- Reported barriers and facilitators to adherence to study product; returning used applicators, and retention in the study
- Gestational age at delivery and birthweight
- Rates of stillbirth
- Serious adverse events and events resulting in study product discontinuation

1.6 ETHICAL ISSUES

The protocol, informed consent documents, and any subsequent modifications will be reviewed and approved by all relevant ethics committees responsible for oversight of the study. For this study, relevant ethics committees include the University of Zambia Biomedical Research Ethics Committee and the University of North Carolina at Chapel Hill Institutional Review Board.

Participation in this trial will be voluntary. All participants will provide written, informed consent prior to study enrolment. All care and procedures will be conducted according to local standards of routine clinical care. All staff who have contact with participants will receive training in the protection of human research participants prior to conducting any study activities and routinely thereafter. Key staff will also complete routine Good Clinical Practices training.

We expect participant risk (detailed in METHODOLOGY below) to be minimal. Progesterone supplementation is used routinely by women at high risk of PTB in the United States and

Europe. The results of this study will enable our research team to determine whether a full-scale efficacy trial of VP for the prevention of PTB among HIV-infected women will be feasible, and how best to support its success through strategies to optimize participant recruitment, adherence, and retention. The knowledge gained from this study and subsequent studies could help to influence health policy that could prevent thousands of preterm births per year in Zambia alone and is expected to outweigh the risks of participation.

2.0 LITERATURE REVIEW

An estimated 15 million babies are born prior to 37 weeks of gestation every year, one million of whom die as a direct result of their prematurity.[8] Over 60% of the world's preterm deliveries occur in Africa and South Asia; in sub-Saharan Africa the rate of preterm birth approaches 20%, compared to rates less than 10% in the Global North.[1] The survival of premature infants depends heavily upon where a baby is born: PTBs in low- and middle-income countries are much more likely to result in neonatal mortality due to unequal access to life-saving technologies and inadequate basic neonatal care. Beyond mortality, lifelong complications of prematurity include neurodevelopmental disorders (e.g. cerebral palsy), vision and hearing loss, learning impairment, and other chronic diseases.[9] Global funding and research has focused largely on developing new technologies to improve outcomes of the preterm infant, much of which is currently applicable only in North America and Europe. Of the strategies that have been studied for PTB prevention, few have proved effective, especially in low- and middle-income countries.[10] Low-cost, high-impact interventions are desperately needed to prevent PTB among those at highest risk for prematurity and its consequences.

Two large meta-analyses performed nearly two decades apart have each shown that HIV-infected women have higher rates of PTB compared to uninfected women.[2,3] The more recent analysis, which included 35 studies through 2014, reported a pooled risk ratio (RR) of PTB among HIV-infected women (not on ART) to be 1.5 times that of uninfected women (RR 1.50; 95% CI: 1.24, 1.82).[3] The strongest and most consistent effect was noted in sub-Saharan Africa, where some 85% of HIV-infected pregnant women reside.[11] HIV-infected women taking ART, with[12-14] or without[15-17] protease inhibitors, experience even higher rates of PTB when compared to HIV-infected, untreated women.[18-26]

Prenatal progesterone – which can be administered intramuscularly or vaginally – reduces the risk of PTB in women who have had a prior spontaneous PTB and in those with evidence of cervical shortening via mid-trimester sonography.[5,7,27,28] Daily vaginal progesterone, in gel or 200-mg suppositories, is recommended as standard of care by the Society of Maternal Fetal Medicine to reduce the risk of PTB among women with ultrasound-identified shortened cervical length.[29] A 2013 Cochrane meta-analysis of trials of VP among women with a prior PTB found the drug to reduce the risk of birth before 37 weeks by half (RR = 0.52; 95% CI: 0.29, 0.92) compared to placebo.[30] Four of these studies[28,31-33] also reported the more severe outcome of PTB before 34 weeks and the treatment effect was even stronger (RR = 0.21; 95% CI: 0.10, 0.44). For the indication of short cervix, a separate meta-analysis[34] of 5 trials reported a VP-attributable reduction in the risk of PTB <35 weeks (RR 0.69, 95% CI 0.55–0.88), <33 weeks (RR 0.58, 95% CI 0.42–0.80), and <28 weeks (RR 0.50, 95% CI 0.30–0.81), as well as lower composite neonatal morbidity and mortality. VP is standard of care in the United States for women with mid-trimester sonographic evidence of shortened cervix.[29,35]

The initiation of labor – whether term or preterm – is an inflammatory event[36] and progesterone has potent anti-inflammatory properties.[36,37] Progesterone suppresses inflammatory cytokines and prostaglandins that induce uterine myocyte contraction, weaken the membranes, and promote cervical remodeling/ softening.[38-42] Progesterone receptor antagonists induce labor in animal models, an effect that can be reversed or delayed with add-back progesterone.[43,44] Declining concentrations of circulating progesterone and reduced progesterone activity are associated with preterm labor.[45] With supplementation, higher serum concentrations of progesterone have incremental protection against preterm labor.[46] HIV infection causes immune activation and inflammation, both systemically and in the lower genital tract.[47,48] While treatment with suppressive ART generally improves systemic inflammation, its effect on the lower genital tract is not nearly as consistent.[49-51] Indeed, HIV-infection is associated with bacterial vaginosis,[52-54] an alteration of the vaginal microbiome that predisposes to PTB[55,56] and that may not be resolved with ART.[57] Further, in the weeks following ART initiation, many women have increased shedding of viral pathogens, such as HSV and CMV in the vagina.[58,59]

Adherence to vaginal study product has been reported as low as 25% in some trials of vaginal microbicides / pre-exposure prophylaxis (PrEP) for HIV prevention in sub-Saharan Africa, rendering primary results from the VOICE and FACTS 001 trials difficult to interpret.[60,61] Poor adherence to vaginal antiretroviral prophylaxis has been associated with a variety of socio-cultural factors, individual participant motivation, and study design.[60,62] In particular, use of a placebo control may diminish participant motivation to use product due to perceived placebo assignment, as could general concerns of participating in the study of an intervention with unproven efficacy.[62] And while adherence has been improving in more recent studies of oral and vaginal PrEP amidst growing evidence of its safety and efficacy,[63-66] a number of concerns remain. In light of these findings, it is prudent to investigate whether adequate adherence can be achieved with VP prior to launching a full-scale efficacy trial.

3.0 METHODOLOGY

3.1 Study design

This will be a mixed method study to evaluate the feasibility and acceptability of a trial of VP to prevent PTB among HIV-infected Zambian women. To assess the *feasibility* of a full-scale clinical trial, we will implement a pilot two-arm, double-masked, placebo-controlled trial of VP among HIV-infected women in antenatal care in Lusaka, Zambia. Participants will be randomly assigned to either daily self-administered VP or indistinguishable placebo commenced after 20 weeks gestation but prior to 24 weeks gestational age. In this pilot study, we will be able to estimate study uptake, adherence to study product and protocol, and study retention. To assess the *acceptability* of a trial to test VP among HIV-infected women in Zambia, we will employ a qualitative approach of longitudinal semi-structured interviews among women agreeing to trial participation and one-time SSIs among those who decline to participate.

3.2 Study sites and study population

This study will be conducted in the antenatal clinics of Kamwala District Clinic (KDC) in Lusaka.

Inclusion criteria:

1. 18 years of age or older

2. viable intrauterine pregnancy confirmed by ultrasound
3. presentation to antenatal care prior to 24 weeks gestation
4. antibody-confirmed HIV-1 infection
5. initiating or continuing ART treatment in pregnancy
6. ability and willingness to provide written informed consent
7. willing to adhere to study visit schedule

Exclusion criteria:
1. multiple gestation
2. non-research indication for antenatal progesterone (i.e. prior spontaneous PTB and/or cervical length <20mm on screening ultrasound)
3. planned or *in situ* cervical cerclage
4. evidence of threatened abortion, preterm labor, or ruptured membranes
5. planned delivery prior to 37 weeks (e.g. prior classical cesarean section)
6. major fetal anomaly detected on screening ultrasound
7. known uterine anomaly
8. known or suspected allergy or contraindication to VP or placebo components

3.3 Study intervention

Pilot randomized controlled trial

Participants will be randomized 1:1 to one of two arms in the pilot randomized controlled trial. The first arm (VP arm) will self-administer daily vaginal progesterone (in 200mg suppositories micronized progesterone) while the other arm (control arm) will self-administer indistinguishable placebo.

Qualitative activities

Two groups of participants will be eligible for participation in the qualitative activities of this study. Women who decline enrollment into the randomized trial will be invited to participate in a one-time face-to-face SSI, while women enrolled and randomized into the placebo-controlled trial will be randomly selected to be followed longitudinally with three serial SSIs.

Study Drug Pharmacological Information

Pharmacologic Category: Micronized progesterone

Chemistry: Micronized progesterone is active by oral and vaginal routes of administration. It is a white or off-white, odorless, crystalline powder that is stable in air and melts between 126°C and 131°C. It is soluble in alcohol, acetone and dioxane; sparingly soluble in vegetable oils; and practically insoluble in water. The chemical name for micronized progesterone is pregn-4-ene-3,20-dione.

Mechanism of Action: Progesterone is a naturally occurring steroid that is secreted by the ovary, placenta, and adrenal gland. In the presence of adequate estrogen, progesterone transforms a proliferative endometrium into a secretory endometrium. Progesterone is necessary to increase endometrial receptivity for implantation of an embryo. Once an embryo is implanted, progesterone acts to maintain a pregnancy. Progesterone is also a potent anti-inflammatory and is thought to prevent preterm birth by counteracting inflammatory processes that lead to cervical remodeling, amniotic membrane rupture and the initiation of

parturition. It is standard of care and recommended for the prevention of preterm birth by the Society for Maternal Fetal Medicine for women who have had a prior spontaneous preterm birth or those with shortened cervical length detected on ultrasound.

Formulation: Vaginal suppository
Strength: 200mg

3.4 Study procedures

Screening

Our recruitment activities will begin with community sensitization in the catchment areas surrounding KDC about the importance of early antenatal care and risks for preterm birth. Trained study staff will conduct health talks at the study sites, focusing on the importance of antenatal care and possible prevention of preterm or early delivery, with emphasis on study inclusion and exclusion criteria. Staff will provide interested women with additional information and referrals to the study clinic for eligibility assessment. Potential participants may also be identified by healthcare workers in the district clinic and referred to the study clinic for screening and enrollment.

After the information session, those interested in participation will be asked to sign a brief consent form for the ultrasound procedure based on a standardized consent for procedures used in government clinics in Zambia. Prior to obtaining this consent, study staff will explain the purpose, benefits, and risks of the ultrasound and the procedures that will be performed.

An ultrasound will be performed for gestational age confirmation and for cervical length measurement to ensure the participant does not meet the exclusion criterion of short cervical length (<20 mm). If the screening process occurs prior to 16 weeks (before a cervical length assessment can be performed), a second ultrasound will be performed at ≥16 weeks' gestation prior to randomization to ensure continued eligibility for study drug. In the rare cases that a short cervical length is encountered after initial screening and enrollment (but prior to randomization), the participant will be offered active progesterone supplementation and will continue to be followed in the study.

Following the ultrasound, all women who meet gestational age and cervical length inclusion criteria will be asked to provide written informed consent for study participation in English, Nyanja, or Bemba. Women who are eligible but decline study enrollment will be invited to participate in a one-time face-to-face semi-structured interview (see QUALITATIVE ACTIVITIES, below), for which they will undergo a separate informed consent process.

Study staff will verify antenatal and HIV history data from participants' antenatal cards and HIV clinic charts for those undergoing screening for the pilot study. In addition, we will perform the following screening tests at the screening visit: hemoglobin, urinalysis, and rapid syphilis. In the case of abnormal screening results, our study clinicians will either provide the necessary medication (i.e. iron, folate, antibiotic) per standard of care, or provide a prescription for the participant to obtain these medications. Additionally, a rapid HIV test will be performed to confirm HIV infection eligibility.

Study product preparation, randomization, and dispensation

Women will undergo randomization into one of two study arms between 20 0/7 and 23 6/7

gestational weeks. The trial will use a paper-based system of sealed envelopes to assign women 1:1 to active drug or placebo. A statistician from the UNC Center for AIDS Research Biostatistics Core not otherwise associated with the study will design the scheme using random permuted blocks.

Study product will be produced and packaged in Chapel Hill, NC. Each package will have an identifying code that will be indexed in a secured database as either placebo or product. Packaged product will be shipped to Zambia and dispensed by on-site pharmacy staff. Participants will be instructed to begin daily self-administration of study product from the day of randomization until 36 6/7 gestational weeks, membrane rupture, or delivery, whichever is sooner. Women will be instructed on correct product use at the enrollment visit and at additional study visits, as needed. Study product will typically be dispensed with single-use applicators at 2- week intervals with an additional 14-day buffer to be replenished as needed at subsequent study visits. Each participant will receive a discreet carrier and plastic bags to facilitate return of used applicators at follow-up visits. Participants will be asked to complete dose diary to facilitate a self-reported adherence measure.

Antenatal, intrapartum, and immediate postpartum study follow-up

Participants will be asked to return to clinic for bi-weekly pharmacy visits with all of their used and unused study product and their dose diaries. The on-site pharmacist will collect used applicators, count unused product, and review participant dose diaries. Trained technicians will then test all returned applicators for evidence of vaginal insertion using a dye stain assay (see LABORATORY PROCEDURES, below). Adherence counseling will be provided to all participants at study initiation and then will be preferentially tailored to participants whose adherence diary falls below 80%. All adherence counseling activities will be provided by trained study staff.

Routine antenatal and HIV care will be provided to all participants by the Zambian district health center (KDC) following national guidelines. Study staff will conduct follow-up visits at approximately 24, 28, 32, and 36 weeks' gestation to gather interval medical and behavioral data from the participant and from her antenatal card. A final ultrasound exam will be performed at 32 weeks' gestation for fetal growth assessment. Our study staff will work with participants to ensure follow-up visits coincide with scheduled antenatal visits as often as possible to reduce burden on participants with regards to time and travel.

Each participant will be asked where she intends to deliver to assist in collection of data on delivery outcomes. For women who deliver in an area clinic or hospital, the study team will obtain detailed information about the clinical management of the participant's delivery, as well as the delivery outcome for both the mother and her infant from medical records. For those who deliver either at home or a distant clinic or hospital, as much delivery information as possible (e.g., date of delivery, infant vital status and birthweight) will be obtained from the participant herself after delivery.

Finally, all participants presenting to the 36-week study visit will be asked to complete a short exit survey.

Visit procedures are summarized in the Schedule of Evaluations (Table 1).

Table 1. Schedule of Study Visits and Procedures

	Scrng	Rand	Follow-up								Final
**Visit Number**	**1.0**	**2.0**	**2.1**	**3.0**	**4.0**	**5.0**	**6.0**	**7.0**	**8.0**	**9.0**	**10.0 Delivery**
**Gestational Age, in weeks**	**≤ $23^{6}/_{7}$**	**$20^{0}/_{7}$- $23^{6}/_{7}$**	**22**	**24**	**26**	**28**	**30**	**32**	**34**	**36**	
**ENROLMENT EVENTS:**											
Eligibility screen	♦										
Informed consent	♦										
Screening ultrasound[1]	♦	♦									
Randomization[2]		♦									
Hemoglobin, urinalysis, rapid HIV & syphilis	♦										
**QUALITATIVE:**											
SSI among decliners	♦										
Longitudinal SSI among study participants	♦						♦			♦	
**STUDY VISITS:**											
Self-administration of study product		♦	♦	♦	♦	♦	♦	♦	♦	♦	
Study product pick-up		♦	♦	♦	♦	♦	♦	♦	♦		
Applicator return			♦	♦	♦	♦	♦	♦	♦	♦	
Collection of medical and behavioral data	♦	♦		♦		♦		♦		♦	♦
Ultrasound for fetal growth								♦			
**ASSESSMENTS:**											
Adherence assessments			♦	♦	♦	♦	♦	♦	♦	♦	
Exit Survey										♦	
Determination of delivery information and infant vital status											♦

1 Screening ultrasound will take place as early as possible on the first study visit prior to 24 weeks. If the screening process occurs prior to 16 $^{0}/_{7}$ weeks, a second ultrasound to measure cervical length will be performed before randomization

2 Participants may be enrolled at any time prior to 24 weeks gestation, but randomization will occur no earlier than 20 $^{0}/_{7}$

Retention

Once a participant is enrolled in the trial, the study team will make reasonable efforts to retain her in follow-up to minimize bias associated with loss to follow-up. The study team will track retention rates and address any issues related to retention. Strategies may include:

- Thorough explanation of the study visit schedule and procedures during informed consent, and re-emphasis at each study visit.
- Encouragement of participants to discuss potential study participation with their husbands/partners and other influential family members before agreeing to enrol in the study.
- Collection of detailed locator information at screening, and review and updating of this

information at each study visit.
- Use of appropriate and timely visit reminder mechanisms (including phone calls and text messages, if participants specifically agree).
- Follow-up on missed visits, including home or other off-site visits if agreed upon.
- Mobilization of trained outreach workers to complete in-person contact with participants at their homes and/or other locations.

Qualitative activities

Women who decline enrollment into the randomized trial will be invited to participate in a one-time, face-to-face SSI to assess the following subject areas: (1) perceived risk of PTB; (2) health beliefs about and attitudes towards HIV, ART and PTB, and towards a medication for the prevention of PTB; (3) acceptability of a daily vaginal medication (as opposed to weekly IM injections); and (4) attitudes towards participation in a double- masked trial that uses a placebo control. We aim to sequentially interview at least 30 women who decline enrollment, with the goal of thematic saturation of data.

Additionally, women who are enrolled into the RCT will be randomly selected to be followed longitudinally with three serial SSIs, to be held at enrollment, mid-study (28-32 weeks' gestational age), and at the final study visit. We aim to conduct interviews with women enrolled in the study to address the same domains outlined above, in addition to barriers and facilitators to: (1) adherence to study product; (2) returning used applicators – i.e. protocol adherence; and (3) retention in the study. If our random sample does not include women from the full range of adherence levels, we will apply purposeful sampling to capture women of underrepresented adherence levels for a single interview in their 3rd trimester. Our expected sample size for this group will be 30 women, based on expectations regarding saturation of qualitative themes.

All interview sessions will be facilitated by a staff member trained in qualitative data collection techniques, in a private location on-site and are expected to last 30 minutes each. Sessions will be audio-recorded and transcribed, with approval provided by participants via informed consent. Where translations are needed, we will have independent reviewers review to ensure accuracy.

3.5 Biological specimen collection

Vaginal study product (progesterone or placebo) will be provided to participants with single-use vaginal applicators at each study visit. Participants will be asked to return all used and unused applicators to each study visit, at which point applicators will be counted and self-reported adherence will be assessed. All returned vaginal applicators will then be tested by trained technicians for evidence of vaginal insertion using standard operating procedures and according to dye stain assay (DSA) specifications (see LABORATORY PROCEDURES, below).[67] Returned applicators will then be disposed of after testing and recording of DSA results. No additional specimens will be collected, with the exception of finger stick blood and urine for hemoglobin, urinalysis, rapid syphilis and HIV confirmation testing at screening. No biological specimens will be stored for the purposes of this study.

3.6 Laboratory procedures

Trained laboratory technicians will test returned applicators using an inert dilute food dye (0.05% FD&C Blue No. 1) that produces a distinctive streaked color when applied via spray

to vaginal applicators after vaginal insertion. Sensitivity and specificity of DSA for vaginal insertion is reported as 97% and 79%, respectively. The DSA has been validated in sub-Saharan Africa, has been accurate at time points up to 4 months after insertion, and has shown no evidence of cross-contamination even when used and unused applicators were commingled in the same bag.[68]

3.7 Data security and management

Data collected on each participant will include sociodemographic information, relevant HIV and obstetrical history, and longitudinal clinical details of current pregnancy and delivery. We will also collect data on adherence to study protocol and study product, and qualitative data from semi-structured interviews.

Study data management (e.g. data transmission, query resolution, etc.) will follow site data management SOPs. Study identification numbers will be used on all forms, applicator storage bags, and communications related to the study. Applicator storage bags will be labeled at the time of dispersal and then, when returned, applicators will be processed via DSA as described above.

A separate confidential register will link study identification numbers and participants' names. All data instruments and registers will be securely stored. Data will be entered into a custom built database and will be validated. Computers will be password protected and their access restricted to authorized study personnel. Backups of the data will be made on a weekly basis. Data may be transmitted electronically to the study investigators through secure cloud-based servers. Study information will not be released without written permission of the participant, except when necessary for monitoring by the relevant ethical committees or their designees.

Data will be disposed of after completion of the study following sponsor guidelines. At that time, electronic records, including linkage codes and identifiers, will be permanently deleted. Paper records will be shredded prior to disposal.

3.8 Statistical considerations

Pilot randomized controlled trial

The primary objective of this research is to determine whether a phase III efficacy trial would be feasible. As such, we will study 3 key indices of feasibility: uptake, adherence, and retention. Given recent concerning results around poor patient adherence to vaginal study product in HIV prevention studies, we have chosen adherence as our primary outcome. The minimum rate of adherence necessary to derive benefit from VP is not known. To keep this work in line with other published work, we will define adequate adherence as a binary variable that describes whether or not a participant has used at least 80% of prescribed study product. We will assess feasibility of a phase III trial with the following criteria for marking success:

1. At least **70% of participants achieve adequate adherence** (i.e., $\geq$70% of women take $\geq$80% of prescribed doses). Since the study is masked to treatment group, we will utilize adherence data from all participants for this assessment (although in an exploratory analysis we will investigate whether adherence differs by treatment group). We used the normal approximation confidence limit approach to choose the sample size for this study,

with the exact binomial CI as a sensitivity analysis. Using a 95% CI for the proportion of randomized patients with adequate adherence and assuming an observed adherence rate of 80%, n=140 provides a lower confidence limit above 70%. The target proportion of women achieving adequate adherence is 70-95%. TABLE 2 presents the precision achieved for outcomes with an observed proportion of 50-95% given a total sample size of 140.

2. At least 90% of participants complete the study. The primary outcome of a VP phase III trial will almost certainly be a composite of PTB and stillbirth. Successful retention in a trial will be defined by the proportion of women in whom we are able to ascertain both the date of delivery and infant vital status. For the present feasibility study, we will require a study visit to define retention success (either at delivery or postpartum), but will also explore whether this outcome can be reliably obtained through tracing patients and querying outcomes via home visits or telephone calls.
3. At least 50% of eligible participants agree to be enrolled. In light of concerns of study participants in sub-Saharan Africa around placebo controls and from our estimates from previous trials in Zambia,[69-72] we believe this level of trial uptake would be feasible. The outcome will be calculated as the proportion of women deemed eligible for enrollment at initial screening who are ultimately randomized.

Table 2: Precision of observed proportions with n=140

	**Observed proportion (adherence, uptake, retention)**					
	**0.5**	**0.6**	**0.7**	**0.8**	**0.9**	**0.95**
**Normal Approximation of Precision**	± 0.083	± 0.081	± 0.076	± 0.066	± 0.050	± 0.036
**Exact binomial CI**	0.41, 0.59	0.51, 0.68	0.62, 0.77	**0.72, 0.86**	0.84, 0.94	0.90, 0.98

Our primary outcome is the proportion of women with adequate adherence, a binary variable that indicates the return of ≥80% of provided vaginal applicators with DSA positivity (unreturned applicators will be considered unused in the primary analysis). As secondary measures, we will also estimate study drug adherence by dose diary and verbal self-report, and compare these techniques to the gold standard DSA to gain insight into the potential utility of these simpler methods in the larger trial. As is evident in TABLE 2, above, our proposed sample size will provide reasonable precision around this estimate. We will also describe the continuous distribution of the percentage of returned vaginal applicators.

In a secondary analysis, we will investigate whether the use of a pre-randomization placebo run-in period would be valuable in a full-scale efficacy trial. Several perinatal trials including multiple progesterone studies have used a placebo run-in period to select participants who are most likely to be adherent prior to randomization. For a phase III VP trial, one could imagine a 2-week run-in period, where women are provided placebo/inert study product and only those returning applicators that met a certain adherence threshold would be randomized. While we do not intend to explicitly pilot this procedure, we will simulate it by dividing the adherence dataset into the first 2 weeks versus the remaining weeks, and evaluate whether some initial threshold adherence (e.g., missing no more than 1 dose) is predictive of adequate adherence over the remaining study period. If non-adherence is seen as the "disease" in a sensitivity/specificity calculation, we might seek the most sensitive cut-point in the run-in period possible, in order to ensure that all non-adherent participants are detected (and exited from the study prior to randomization). Of course, high sensitivity comes at the cost of false

positive results (i.e., disqualifying women who would have been adherent). This exercise will allow us to evaluate the utility of a run-in in the overall context of phase III planning, when issues such as recruitment and representativeness are also being considered. Statistical approaches for diagnostic testing such as ROC curve analyses (plot of sensitivity versus 1-specificity) will be used to inform selection of the non-adherence cut-point. Sensitivity, specificity, PPV and NPV of the chosen cut-point will be estimated with a corresponding 95% exact binomial confidence interval.

We will define uptake strictly as the proportion of women meeting initial (screening) eligibility criteria who are randomized in the trial. We will also measure (1) the proportion of ANC attendees who meet initial eligibility criteria, and (2) the proportion of those meeting initial eligibility criteria who are eventually deemed ineligible (e.g., after ultrasound). If uptake in the pilot is very low, enrollment of a large trial might not be feasible. Low uptake might also make us worry about external validity and whether the trial would be representative enough to be of real policy value.

To quantify study retention, we will calculate the proportion of women randomized in the trial for whom we are able to ascertain the date of delivery and infant vital status at birth. For the present study, we will require a clinic visit to define retention success (either at delivery or postpartum), but will also explore whether this outcome can be reliably obtained through home visit or even a telephone call. Based on prior experience, we expect this rate to exceed 90%. Assuming the observed retention proportion is 95%, our sample size of 140 patients will provide a 95% CI spanning 90-98% (TABLE 2).

Univariate analyses will be performed to identify maternal demographic and health characteristics associated with adherence, uptake and retention; and adjusted associations will be analyzed with a multivariable log-binomial model as appropriate (for outcomes with a sufficient number of events).

Secondary efficacy and safety outcomes will include: (a) delivery prior to 37 weeks gestation; (b) delivery prior to 34 weeks gestation; (c) birth weight <2500g; (d) stillbirth; and (e) adverse events. We will attempt to trace all participants who are lost to follow-up to determine obstetrical and neonatal outcomes. In an exploratory analysis of these outcomes, we will conduct descriptive statistics of participant demographic and clinical features, and of the proportion of patients experiencing the secondary outcomes of poor obstetrical and neonatal outcomes. Although our study is not powered for efficacy, we will perform univariate analyses to determine if a numerical difference in risk of these secondary efficacy and safety outcomes exists between women who were randomized to VP compared to those randomized to placebo. Sensitivity analyses will be used to investigate how limiting the analysis to high adherers impacts the estimated RR. The effect of participant demographic variables (and any additional potential measured confounders) will be explored in multivariable analyses using a log-binomial model. Exploratory analyses will be stated as such and presented as pilot effect estimates with a 95% CI; such analyses will provide useful results for planning a full-scale trial.

In sum, we expect this feasibility study to allow us to estimate the proportion of women who (a) are eligible to participate, (b) will agree to participate, (c) are able to comply with the visit schedule, (d) will return with used applicators, (e) will adhere to prescribed study drug, and (f) will remain in the study once enrolled. We will gain insight into the (g) baseline event rate, (h) preliminary effect size, and (i) rates of adverse events. Finally, the study will allow us to develop and test study-specific SOPs and gain experience with randomization.

Qualitative activities

Our primary objective is to identify common facilitators and barriers to trial uptake, vaginal product adherence, and retention in a clinical trial. We will first analyze data from women who decline enrollment independently from data among those who enroll. Then, findings from common baseline domains evaluated in both assessment groups will be compared to identify commonalities or discrepancies between decliners and enrollees. The longitudinal data assessed from interviews with enrolled participants will be compared across time and between those randomized to placebo versus progesterone. After translations and transcription, a team of coders with qualitative data analysis experience will use qualitative analysis software to organize the transcribed data. Transcript data will initially be organized based on the questions in the semi-structured guides. Coders will read and "memo" each transcript and compare memos to create a preliminary codebook. They will apply the codes to significant utterances, comparing their results periodically (such as every 4-5 interviews) to assess consistency between coders in order to refine a final codebook. They will examine codes and utterances for clusters of meaning to construct themes and maps reflecting categories and relationships among codes. They will then examine the themes identified from each dataset for contrasts and overlaps between them, which may lead to reconstructions of the existing themes or the emergence of new subthemes. A summary report of the analyzed SSI data will be created which will inform the development of the following materials for the planned full-scale efficacy and safety trial: study protocol, consent forms, adherence monitoring and counseling guide, and provider training materials.

3.9 Human participant considerations

As described in Section 1.6, the protocol, informed consent, and all participant materials will be reviewed by relevant ethics committees maintaining Federal Wide Assurances (FWA) with Office for Human Research Protections (OHRP) approval. All staff who have contact with participants will receive training on the protection of human research participants prior to conducting any study activities and routinely thereafter.

Informed consent

Discussions with prospective participants and informed consent procedures will be conducted in private to protect patient confidentiality. Where possible, a private room will be used to discuss the study and potential participant's eligibility. If a private room is not available, a designated area far enough away from other patients such that they cannot hear the conversation will be used. The study nurse will obtain written informed consent from all participants. The study procedures, risks, and benefits will be discussed and the study nurse will answer all questions prior to obtaining consent. The consent forms will be translated into local languages (Nyanja and Bemba) and back-translated into English to assure accurate translation. All versions of the consent forms will be approved by the relevant ethics committees prior to study initiation. For illiterate participants, a literate impartial witness will be present during the entire consent process to ensure that all of the relevant information has been provided and the participant voluntarily gives consent.

Eligible women who do not wish to participate in this study will continue to receive antenatal care and treatment according to local clinical standards.

Potential risks to participants

Investigators will make efforts to minimize risks to participants. It is expected that this study will expose subjects to minimal risks. Side effects and serious adverse events with vaginal progesterone administration are very rare (see ADVERSE EVENTS, below). Self-administration of vaginal progesterone may cause mild discomfort to participants. This risk of discomfort to participants will be minimized by training of participants in correct use of vaginal applicators by study staff.

Participation in clinical research includes the risks of loss of confidentiality and discomfort with the personal nature of questions, particularly when discussing HIV infection or sexual behaviors. At each step in the study, we will protect participant privacy and confidentiality to reduce these risks. Although investigators make every effort to protect participant privacy and confidentiality, it is possible that participant involvement in the study could become known to others, and that social harms may result (i.e. as participants could become known as HIV infected).

The confidentiality of all study records will be safeguarded to the extent legally possible. To maintain participant confidentiality, all laboratory specimens, reports, study data and administrative forms will be identified by a coded number only. All databases will be secured with password-protected access systems, and computer entries will be identified by coded number only. Forms, lists, logbooks, appointment books, and any other listings or data forms that link participant ID numbers to other identifying information will be stored in a separate, locked cabinet. All data analysis will be performed using datasets which have only the study number as a unique identifier. Clinical information with individual identifiers will not be released without the written permission of the participant. We expect these procedures to adequately protect participant confidentiality.

Potential benefits to participants and others

Individual participants in the VP arm of the randomized trial may benefit from a reduction in preterm birth risk if the intervention is found to be effective, but it is also possible that there will be no effect. We believe that equipoise exists between the study arms since VP has not been previously studied in this population of HIV infected women receiving antiretroviral therapy in sub-Saharan Africa, and the exact risk for preterm birth in this population remains unclear. All participants may benefit from enhanced health education, and close clinical monitoring. Knowledge generated from this study has the potential to inform future clinical trials on the reduction of PTB risk among HIV-infected women, which may enable policymakers worldwide to make informed decisions regarding effective interventions for the prevention of PTB.

At the clinic level, all staff involved in the study will receive refresher training on the national guidelines for the diagnosis and treatment of preterm birth. Increased awareness about screening and treatment for preterm birth may help to improve care overall at KDC. Additionally, our community sensitization activities may help to encourage more women to come for antenatal screening earlier in pregnancy, which has a known benefit on maternal and infant outcomes.

In summary, we expect participant risk to be minimal. The knowledge generated from this study regarding the potential PTB risk reduction among HIV-infected women is expected to outweigh the risks of participation.

Inclusion of children, sub-populations, and vulnerable populations

As this is a trial specifically investigating an intervention for prevention of preterm birth among HIV-infected women 18 years old or older, only eligible women will be recruited to participate.

3.10 Safety monitoring

At each study visit, study staff will evaluate participants for social harms and adverse events (AEs). A social harm will be defined as a non-medical untoward consequence of study participation, including: difficulties in personal relationships, stigma, or discrimination from family or community. An AE will be defined as any untoward medical occurrence in a study participant including any abnormal sign (e.g., abnormal physical exam or laboratory finding), symptom, or disease, temporally associated with the individual's participation in the research, whether or not considered related to participation in the research. In addition to events related to study procedures and vaginal study product, we can expect that this population of HIV-infected pregnant women to experience adverse events unrelated to study procedures, including adverse obstetrical outcomes, opportunistic infections, side effects from ART or other medications, hospitalization, and death.

The severity of study-related adverse events and social harms will be graded using the National Institute of Health's Division of AIDS Table for Grading the Severity of Adult and Pediatric Adverse Events. We will also record information on all serious adverse events (SAEs) occurring in participants whether or not they are related to study participation or the study drug, including AEs that:

1. Result in hospital admission (unless hospitalization is preplanned, i.e. for delivery) or prolongation of existing hospitalization
2. Are immediately life-threatening, including drug reactions that necessitate discontinuation of study participation,
3. Cause significant, persistent, or permanent harm or disability, either physical or psychological,
4. Result in death, including fetal demise after 20 weeks gestation
5. Are congenital anomalies/birth defects

Information on adverse events or social harms that are related to the study drug and all SAEs will be documented on study data forms and routinely reported to the Principal Investigator or designee. If the PI, co-investigators, or their designees determine that study-related adverse events are occurring at an unexpected rate, they will assess the need for staff re-training, protocol amendment, or study cessation. Adverse Events will be reported to the UNZA REC, UNC IRB, and other regulatory bodies according to their individual guidelines.

3.11 Dissemination of findings

Study findings will be disseminated through appropriate local channels, including academic and public health research symposia. One or more publications will also be submitted to a peer-reviewed journal. Our study team will plan to publish results whether positive or negative. The study participants' privacy and confidentiality will be strictly maintained in all results dissemination or publication activities.

4.0 BUDGET (1 USD = 10 ZMW)

ITEM	USD TOTAL	ZMW TOTAL
Personnel	$200,000	K 2,000,000
Equipment	$0	K 0
Supplies & materials	$31,687	K 316,870
Participant costs	$5,409	K 54,090
Contractual	$0	K 0
Other	$8,855	K 88,550
TOTAL	$245,951	K 2,459,510

5.0 TIMELINE

Participants will be followed from enrollment (prior to 24 weeks gestational age) through delivery. We have allocated 6 months to start-up activities and 3 months to wind-down activities.

PHASE	Year 1				Year 2			
	Q1	Q2	Q3	Q4	Q1	Q2	Q3	Q4
**Pre-implementation**								
Regulatory approvals	X							
Site preparation and training	X	X						
**Implementation**								
Enrollment		X	X	X	X			
Follow-up			X	X	X	X	X	X
**Data management, analysis & reporting**								
Transcription and coding of interviews			X	X	X	X		
Data entry and management		X	X	X	X	X	X	X
Statistical analysis					X	X	X	X
Publication and dissemination								X

6.0 REFERENCE LIST

1. Blencowe H, Cousens S, Oestergaard MZ, et al. National, regional, and worldwide estimates of preterm birth rates in the year 2010 with time trends since 1990 for selected countries: a systematic analysis and implications. *The Lancet.* 2012;379(9832):2162-2172.
2. Brocklehurst P. The Association between maternal HIV infection and perinatal outcome: a systematic review of the literature and meta-analysis. *Br J Obstet Gynaecol.*105:836-848.
3. Wedi CO, Kirtley S, Hopewell S, Corrigan R, Kennedy SH, Hemelaar J. Perinatal outcomes associated with maternal HIV infection: a systematic review and meta-analysis. *Lancet HIV.* 2016;3(1):e33-48.
4. Fowler MG, Qin M., Fiscus S.A., Currier J.S., Makanani B,. Martinson F., Chipato T., Browning R., Shapiro D., Mofenson L. PROMISE: Efficacy and safety of 2 strategies to prevent perinatal HIV transmission. Conference on Retroviruses and Opportunistic Infections; February 23-24, 2015; Seattle.
5. Hassan SS, Romero R, Vidyadhari D, et al. Vaginal progesterone reduces the rate of preterm birth in women with a sonographic short cervix: a multicenter, randomized, double-blind, placebo-controlled trial. *Ultrasound Obstet Gynecol.* 2011;38(1):18-31.

6. da Fonseca EB, Bittar RE, Carvalho MHB, Zugaib M. Prophylactic administration of progesterone by vaginal suppository to reduce the incidence of spontaneous preterm birth in women at increased risk: A randomized placebo-controlled double-blind study. *American Journal of Obstetrics and Gynecology.* 2003;188(2):419-424.
7. Fonseca EB, Celik E, Parra M, Singh M, Nicolaides KH, Fetal Medicine Foundation Second Trimester Screening G. Progesterone and the risk of preterm birth among women with a short cervix. *N Engl J Med.* 2007;357(5):462-469.
8. March of Dimes P, Save the Children, WHO. *Born too Soon: the Global Action Report on Preterm Birth.* Geneva: WHO;2012.
9. Hack M, Taylor HG, Drotar D, et al. Chronic conditions, functional limitations, and special health care needs of school-aged children born with extremely low-birth-weight in the 1990s. *JAMA.* 2005;294(3):318-325.
10. Barros FC, Bhutta ZA, Batra M, et al. Global report on preterm birth and stillbirth (3 of 7): evidence for effectiveness of interventions. *BMC Pregnancy Childbirth.* 2010;10 Suppl 1:S3.
11. Joint United Nations Programme on HIV/AIDS. The Gap Report. 2014; http://www.unaids.org/sites/default/files/media_asset/UNAIDS_Gap_report_en.pdf. Accessed March 14, 2016.
12. Chen JY, Ribaudo HJ, Souda S, et al. Highly active antiretroviral therapy and adverse birth outcomes among HIV-infected women in Botswana. *J Infect Dis.* 2012;206(11):1695-1705.
13. Powis KM, Kitch D, Ogwu A, et al. Increased risk of preterm delivery among HIV-infected women randomized to protease versus nucleoside reverse transcriptase inhibitor-based HAART during pregnancy. *J Infect Dis.* 2011;204(4):506-514.
14. Cotter AM, Garcia AG, Duthely ML, Luke B, O'Sullivan MJ. Is antiretroviral therapy during pregnancy associated with an increased risk of preterm delivery, low birth weight, or stillbirth? *The Journal of infectious diseases.* 2006;193(9):1195-1201.
15. Li N, Sando MM, Spiegelman D, et al. Antiretroviral Therapy in Relation to Birth Outcomes among HIV-infected Women: A Cohort Study. *J Infect Dis.* 2016;213(7):1057-1064.
16. van der Merwe K, Hoffman R, Black V, Chersich M, Coovadia A, Rees H. Birth outcomes in South African women receiving highly active antiretroviral therapy: a retrospective observational study. *J Int AIDS Soc.* 2011;14:42.
17. Townsend CL, Cortina-Borja M, Peckham CS, Tookey PA. Antiretroviral therapy and premature delivery in diagnosed HIV-infected women in the United Kingdom and Ireland. *AIDS.* 2007;21(8):1019-1026.
18. Darak S, Darak T, Kulkarni S, et al. Effect of highly active antiretroviral treatment (HAART) during pregnancy on pregnancy outcomes: experiences from a PMTCT program in western India. *AIDS Patient Care STDS.* 2013;27(3):163-170.
19. Townsend C, Schulte J, Thorne C, et al. Antiretroviral therapy and preterm delivery-a pooled analysis of data from the United States and Europe. *BJOG.* 2010;117(11):1399-1410.
20. Machado ES, Hofer CB, Costa TT, et al. Pregnancy outcome in women infected with HIV-1 receiving combination antiretroviral therapy before versus after conception. *Sex Transm Infect.* 2009;85(2):82-87.
21. Koss CA, Natureeba P, Plenty A, et al. Risk factors for preterm birth among HIV-infected pregnant Ugandan women randomized to lopinavir/ritonavir- or efavirenz-based antiretroviral therapy. *J Acquir Immune Defic Syndr.* 2014;67(2):128-135.
22. Lorenzi P, Spicher VM, Laubereau B, et al. Antiretroviral therapies in pregnancy: maternal, fetal and neonatal effects. Swiss HIV Cohort Study, the Swiss Collaborative

HIV and Pregnancy Study, and the Swiss Neonatal HIV Study. *AIDS.* 1998;12(18):F241-247.
23. Martin F, Taylor GP. Increased rates of preterm delivery are associated with the initiation of highly active antiretrovial therapy during pregnancy: a single-center cohort study. *J Infect Dis.* 2007;196(4):558-561.
24. European Collaborative S, Swiss M, Child HIVCS. Combination antiretroviral therapy and duration of pregnancy. *AIDS.* 2000;14(18):2913-2920.
25. Sibiude J, Warszawski J, Tubiana R, et al. Premature delivery in HIV-infected women starting protease inhibitor therapy during pregnancy: role of the ritonavir boost? *Clin Infect Dis.* 2012;54(9):1348-1360.
26. Thorne C, Patel D, Newell ML. Increased risk of adverse pregnancy outcomes in HIV-infected women treated with highly active antiretroviral therapy in Europe. *AIDS.* 2004;18(17):2337-2339.
27. Meis PJ, Klebanoff M, Thom E, et al. Prevention of recurrent preterm delivery by 17 alpha-hydroxyprogesterone caproate. *N Engl J Med.* 2003;348(24):2379-2385.
28. da Fonseca EB, Bittar RE, Carvalho MH, Zugaib M. Prophylactic administration of progesterone by vaginal suppository to reduce the incidence of spontaneous preterm birth in women at increased risk: a randomized placebo-controlled double-blind study. *Am J Obstet Gynecol.* 2003;188(2):419-424.
29. Society for Maternal-Fetal Medicine Publications Committee waoVB. Progesterone and preterm birth prevention: translating clinical trials data into clinical practice. *Am J Obstet Gynecol.* 2012;206(5):376-386.
30. Dodd J, Jones L, Flenady V, Cincotta R, Crowther C. Prenatal administration of progesterone for preventing preterm birth in women considered to be at risk of preterm birth. *Cochrane Database of Systematic Reviews.* 2013(7).
31. Akbari S, Birjandi M, Mohtasham N. Evaluation of the effect of progesterone on prevention of preterm delivery and its complications. *Scientific Journal of Kurdistan University of Medical Sciences.* 2009;14(3):9-11.
32. Cetingoz E, Cam C, Sakalli M, Karateke A, Celik C, Sancak A. Progesterone effects on preterm birth in high-risk pregnancies: a randomized placebo-controlled trial. *Arch Gynecol Obstet.* 2011;283(3):423-429.
33. Majhi P, Bagga R, Kalra J, Sharma M. Intravaginal use of natural micronised progesterone to prevent pre-term birth: a randomised trial in India. *J Obstet Gynaecol.* 2009;29(6):493-498.
34. Romero R, Nicolaides K, Conde-Agudelo A, et al. Vaginal progesterone in women with an asymptomatic sonographic short cervix in the midtrimester decreases preterm delivery and neonatal morbidity: a systematic review and metaanalysis of individual patient data. *Am J Obstet Gynecol.* 2012;206(2):124 e121-119.
35. Committee on Practice Bulletins-Obstetrics TACoO, Gynecologists. Practice bulletin no. 130: prediction and prevention of preterm birth. *Obstet Gynecol.* 2012;120(4):964-973.
36. Rinaldi SF, Hutchinson JL, Rossi AG, Norman JE. Anti-inflammatory mediators as physiological and pharmacological regulators of parturition. *Expert Rev Clin Immunol.* 2011;7(5):675-696.
37. Iams JD, Romero R, Culhane JF, Goldenberg RL. Primary, secondary, and tertiary interventions to reduce the morbidity and mortality of preterm birth. *The Lancet.* 2008;371(9607):164-175.
38. Rubens CE, Sadovsky Y, Muglia L, Gravett MG, Lackritz E, Gravett C. Prevention of preterm birth: Harnessing science to address the global epidemic. *Sci Transl Med.* 2014;6(262):262sr265.

39. Luo G, Abrahams VM, Tadesse S, et al. Progesterone inhibits basal and TNF-alpha-induced apoptosis in fetal membranes: a novel mechanism to explain progesterone-mediated prevention of preterm birth. *Reprod Sci.* 2010;17(6):532-539.
40. Shields AD, Wright J, Paonessa DJ, et al. Progesterone modulation of inflammatory cytokine production in a fetoplacental artery explant model. *Am J Obstet Gynecol.* 2005;193(3 Pt 2):1144-1148.
41. Gotkin JL, Celver J, McNutt P, et al. Progesterone reduces lipopolysaccharide induced interleukin-6 secretion in fetoplacental chorionic arteries, fractionated cord blood, and maternal mononuclear cells. *Am J Obstet Gynecol.* 2006;195(4):1015-1019.
42. Schwartz N, Xue X, Elovitz MA, Dowling O, Metz CN. Progesterone suppresses the fetal inflammatory response ex vivo. *Am J Obstet Gynecol.* 2009;201(2):211 e211-219.
43. Wolf JP, Sinosich M, Anderson TL, Ulmann A, Baulieu EE, Hodgen GD. Progesterone antagonist (RU 486) for cervical dilation, labor induction, and delivery in monkeys: effectiveness in combination with oxytocin. *Am J Obstet Gynecol.* 1989;160(1):45-47.
44. Elovitz MA, Mrinalini C. The use of progestational agents for preterm birth: lessons from a mouse model. *Am J Obstet Gynecol.* 2006;195(4):1004-1010.
45. Mazor M, Hershkowitz R, Ghezzi F, et al. Maternal plasma and amniotic fluid 17 beta-estradiol, progesterone and cortisol concentrations in women with successfully and unsuccessfully treated preterm labor. *Arch Gynecol Obstet.* 1996;258(2):89-96.
46. Caritis SN, Venkataramanan R, Thom E, et al. Relationship between 17-alpha hydroxyprogesterone caproate concentration and spontaneous preterm birth. *Am J Obstet Gynecol.* 2014;210(2):128 e121-126.
47. Appay V, Sauce D. Immune activation and inflammation in HIV-1 infection: causes and consequences. *J Pathol.* 2008;214(2):231-241.
48. Spencer LY, Christiansen S, Wang CH, et al. Systemic Immune Activation and HIV Shedding in the Female Genital Tract. *J Acquir Immune Defic Syndr.* 2016;71(2):155-162.
49. Neely MN, Benning L, Xu J, et al. Cervical shedding of HIV-1 RNA among women with low levels of viremia while receiving highly active antiretroviral therapy. *J Acquir Immune Defic Syndr.* 2007;44(1):38-42.
50. Cu-Uvin S, DeLong AK, Venkatesh KK, et al. Genital tract HIV-1 RNA shedding among women with below detectable plasma viral load. *AIDS.* 2010;24(16):2489-2497.
51. Ondoa P, Gautam R, Rusine J, et al. Twelve-Month Antiretroviral Therapy Suppresses Plasma and Genital Viral Loads but Fails to Alter Genital Levels of Cytokines, in a Cohort of HIV-Infected Rwandan Women. *PLoS One.* 2015;10(5):e0127201.
52. Cohen CR, Lingappa JR, Baeten JM, et al. Bacterial vaginosis associated with increased risk of female-to-male HIV-1 transmission: a prospective cohort analysis among African couples. *PLoS Med.* 2012;9(6):e1001251.
53. Martin HL, Richardson BA, Nyange PM, et al. Vaginal lactobacilli, microbial flora, and risk of human immunodeficiency virus type 1 and sexually transmitted disease acquisition. *J Infect Dis.* 1999;180(6):1863-1868.
54. Myer L, Denny L, Telerant R, Souza M, Wright TC, Jr., Kuhn L. Bacterial vaginosis and susceptibility to HIV infection in South African women: a nested case-control study. *J Infect Dis.* 2005;192(8):1372-1380.

55. Bretelle F, Rozenberg P, Pascal A, et al. High Atopobium vaginae and Gardnerella vaginalis vaginal loads are associated with preterm birth. *Clin Infect Dis.* 2015;60(6):860-867.
56. Meis PJ, Goldenberg RL, Mercer B, et al. The preterm prediction study: significance of vaginal infections. National Institute of Child Health and Human Development Maternal-Fetal Medicine Units Network. *Am J Obstet Gynecol.* 1995;173(4):1231-1235.
57. Warren D, Klein RS, Sobel J, et al. A multicenter study of bacterial vaginosis in women with or at risk for human immunodeficiency virus infection. *Infect Dis Obstet Gynecol.* 2001;9(3):133-141.
58. Gianella S, Redd AD, Grabowski MK, et al. Vaginal Cytomegalovirus Shedding Before and After Initiation of Antiretroviral Therapy in Rakai, Uganda. *J Infect Dis.* 2015;212(6):899-903.
59. Tobian AA, Grabowski MK, Serwadda D, et al. Reactivation of herpes simplex virus type 2 after initiation of antiretroviral therapy. *J Infect Dis.* 2013;208(5):839-846.
60. Marrazzo JM, Ramjee G, Richardson BA, et al. Tenofovir-based preexposure prophylaxis for HIV infection among African women. *N Engl J Med.* 2015;372(6):509-518.
61. Rees H, Delany-Moretlwe, S.A., Lombard, C., Barond, D., Panchia, R., Myer, L., Schwartz, J.L., Doncel, G.F., Gray, G. FACTS 001 Phase III Trial of Pericoital Tenofovir 1% Gel for HIV Prevention in Women. CROI; 2015; Seattle, WA.
62. van der Straten A, Stadler J, Montgomery E, et al. Women's experiences with oral and vaginal pre-exposure prophylaxis: the VOICE-C qualitative study in Johannesburg, South Africa. *PLoS One.* 2014;9(2):e89118.
63. Baeten JM, Palanee-Phillips T, Brown ER, et al. Use of a Vaginal Ring Containing Dapivirine for HIV-1 Prevention in Women. *N Engl J Med.* 2016.
64. Bekker LG, Grant, R., Hughes, J., Roux, S. HPTN 067/ADAPT Cape Town: a comparison of daily and nondaily PrEP dosing in African women. Conference on Retroviruses and Opportunistic Infections; 23-26 February, 2015; Seattle, WA.
65. Baeten J HR, Kidoguchi L, Mugo N, Katabira E, Bukusi E, Asiimwe S, Haberer J, Donnell D, Celum C. Near elimination of HIV transmission in a demonstration project of PrEP and ART. Conference on Retroviruses and Opportunistic Infections; February 23–26, 2015; Seattle, WA.
66. Henderson F.L. TAW, Chirwa L.I., Williams T.S., Borkowf C.B., Kasonde M., Mutanhaurwa R., Matlhaba O., Hageman K., Casillas P., Samandari T. Characteristics and Oral PrEP Adherence in the TDF2 Open-Label Extension in Botswana. International AIDS Society Conference; 18–22 July, 2015; Vancouver, BC.
67. van der Straten A, Cheng H, Mensch B, et al. Evaluation of 3 approaches for assessing adherence to vaginal gel application in clinical trials. *Sex Transm Dis.* 2013;40(12):950-956.
68. Wallace AR, Teitelbaum A, Wan L, et al. Determining the feasibility of utilizing the microbicide applicator compliance assay for use in clinical trials. *Contraception.* 2007;76(1):53-56.
69. Chi BH, Sinkala M, Mbewe F, et al. Single-dose tenofovir and emtricitabine for reduction of viral resistance to non-nucleoside reverse transcriptase inhibitor drugs in women given intrapartum nevirapine for perinatal HIV prevention: an open-label randomised trial. *Lancet.* 2007;370(9600):1698-1705.
70. Goldenberg RL, Mwatha A, Read JS, et al. The HPTN 024 Study: the efficacy of antibiotics to prevent chorioamnionitis and preterm birth. *Am J Obstet Gynecol.* 2006;194(3):650-661.

71. Stringer EM, Kaseba C, Levy J, et al. A randomized trial of the intrauterine contraceptive device vs hormonal contraception in women who are infected with the human immunodeficiency virus. *Am J Obstet Gynecol.* 2007;197(2):144 e141-148.
72. Stringer JS, McConnell MS, Kiarie J, et al. Effectiveness of non-nucleoside reverse-transcriptase inhibitor-based antiretroviral therapy in women previously exposed to a single intrapartum dose of nevirapine: a multi-country, prospective cohort study. *PLoS Med.* 2010;7(2):e1000233.
